# The Army Medical Bill

**Published:** 1908-02

**Authors:** 


### The Army Medical Bill.

THERE is every prospect of the Army Medical reorganisation bill becoming a law during the present session provided the medical profession makes an impression on the members of Congress and urges them to do all in their power to secure action on the measure. If the bill comes to a vote it will assuredly pass. This the JOURNAL can state with a degree of certainty, for there is no question that a majority of the legislators are in favor of the proposed reorganisation of the medical department which has the approval of the War Department and the President.

The committee on Military Affairs of the House reported the bill favorably on January 7, 1908, and the next day similar action was taken by the Senate committee. The bill is thus practically before the House and needs only the permission of the Speaker to receive action. The same bill has been before the House and Senate two years now, and there is nothing in the way of its passage by an almost unanimous vote except the disinclination of the Speaker to permit it to come up for final action.

It is a most necessary bill and the sooner it is allowed to become a law, as it assuredly will so soon as it reaches a vote, the sooner will the Surgeon-General be able to place his department on a numerically efficient basis.

Members of the profession are urged to communicate with the Congressman of their several districts, requesting their hearty coöperation in getting the bill through the present session.

